# Continuously available ratio of SpO_2_/FiO_2_ serves as a noninvasive prognostic marker for intensive care patients with COVID-19

**DOI:** 10.1186/s12931-020-01455-4

**Published:** 2020-07-22

**Authors:** Xiaofan Lu, Liyun Jiang, Taige Chen, Yang Wang, Bing Zhang, Yizhou Hong, Jun Wang, Fangrong Yan

**Affiliations:** 1 State Key Laboratory of Natural Medicines, Research Center of Biostatistics and Computational Pharmacy, China Pharmaceutical University, Nanjing, 210009 China; 2 Department of Biostatistics, The University of Texas MD Anderson Cancer Center, Houston, 77030 TX USA; 3 Medical School of Nanjing University, Nanjing, 210093 China; 4 Department of Radiology, The Affiliated Nanjing Drum Tower Hospital of Nanjing University Medical School, Nanjing, 210008 China; 5 Department of Intensive Care Medicine, The First Affiliated Hospital of Soochow University, No. 188 Shizi Street, Suzhou, 215006 China

**Keywords:** COVID-19, SpO_2_/FiO_2_, Joint model, Prognostic marker

## Abstract

**Rationale:**

Oxygen saturation to fraction of inspired oxygen ratio (SpO_2_/FiO_2_) has been described as potential predictor of poor outcome for COVID-19, without considering its time-varying behavior though.

**Methods:**

Prognostic value of SpO_2_/FiO_2_ was evaluated by jointly modeling the longitudinal responses of SpO_2_/FiO_2_ and time-to-event data retrieved from 280 severe and critically ill (intensive care) patients with COVID-19.

**Results:**

A sharply decrease of SpO_2_/FiO_2_ from the first to second measurement for non-survivors was observed, and a strong association between square root SpO_2_/FiO_2_ and mortality risk was demonstrated, with a unit decrease in the marker corresponding to 1.82-fold increase in mortality risk (95% CI: 1.56–2.13).

**Conclusions:**

The current study suggested that SpO_2_/FiO_2_ could serve as a non-invasive prognostic marker to facilitate early adjustment for treatment, thus improving overall survival.

## Introduction

Epidemic studies have been well described clinical characteristics of patients with coronavirus disease 2019 (COVID-19), with several clinical features being potential predictors of poor outcome, including the oxygen saturation to fraction of inspired oxygen ratio (SpO_2_/FiO_2_) [1]. However, the way potential prognostic factors were identified is far from being informative because it is usually analyzed as a fixed baseline covariate, without considering its time-varying behavior [2]. The purpose of this study is to preliminarily evaluate the prognostic value of SpO_2_/FiO_2_ in the disease management of COVID-19 among intensive care patients within a joint modeling approach, which may allow us to capture and quantify the association between the dynamic measurements of SpO_2_/FiO_2_ and the survival outcome.

## Methods

### Study participants

This study originally enrolled 344 severe and critically ill patients (intensive care patients) who were diagnosed with COVID-19 and were hospitalized in Tongji hospital from January 25 through February 25, 2020. The illness severity of COVID-19 was defined according to the Chinese management guideline for COVID-19 (version 6.0) [3]. The ratio of SpO_2_/FiO_2_ was measured at day 1, 3, 7, 14 and 28 since admission to intensive care wards. Survival endpoint was 28-day mortality after admission. Characteristics of these 344 patients have been detailed described in our previous study [1]. Potential mortality-associated confounders were considered for adjustment according to previous literatures [1, 2], including age, lymphocyte count, and D-Dimer content that were recorded at admission. Respiratory support throughout the disease course was also retrieved due to its effect on SpO_2_/FiO_2_. Specifically, patient was regarded as affirmative respiratory support if received either one of the following treatments: non-invasive or invasive ventilators, high-flow nasal cannula oxygen therapy, and extracorporeal membrane oxygenation. After filtering out patients with any missing data, 280 out of 344 patients were eventually identified for this study. The Ethics Commission of Tongji hospital approved this study, with a waiver of informed consent.

### Statistical analyses

We proceeded by specifying a joint longitudinal-survival model that explicitly accounts for the endogeneity of the SpO_2_/FiO_2_ marker. In particular, we started by fitting a linear mixed-effects sub-model for the longitudinal outcome of SpO_2_/FiO_2_ using *nlme* R package; we included the main effect of time (time points that the corresponding longitudinal response were recorded), respiratory support, and the interaction of treatment with time for the fixed-effects part, and we included an intercept and a time term for the random-effects part. For the survival sub-model, a multivariate Cox proportional hazards regression model was fitted, in which mortality-associated confounders were involved. After having separate sub-models, we jointly modeled the longitudinal responses and time-to-event data under a maximum likelihood approach by using *JM* R package [4].

## Results

**Fig. 1:**
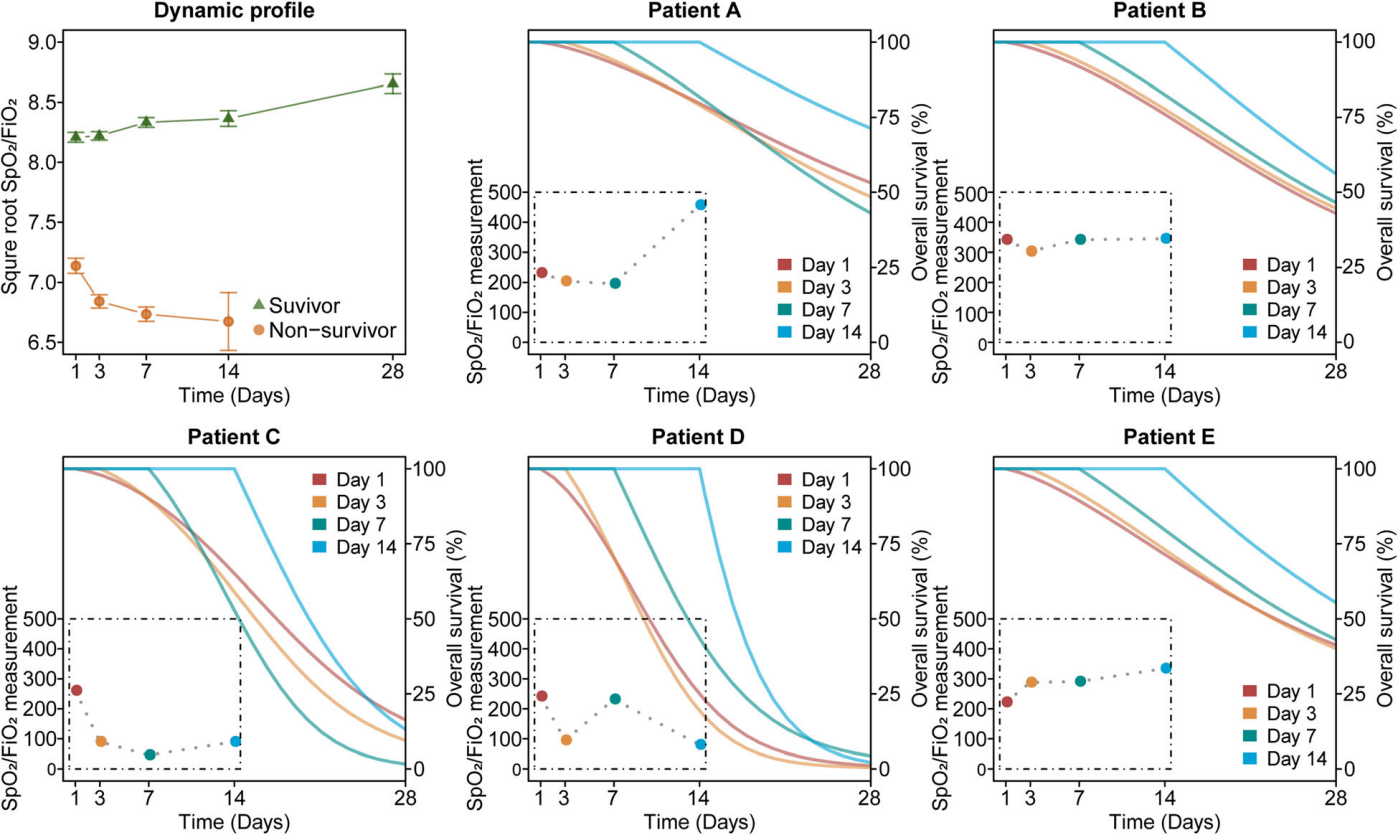
Dynamic profile of SpO_2_/FiO_2_ marker and dynamic survival probabilities of five intensive care patients with COVID-19 during follow-up. The first time line chart illustrates the distribution (mean ± standard error) of square root SpO_2_/FiO_2_ in 280 patients (112 non-survivors and 168 survivors) at each measurement time point, and no record of day 28 for non-survivors because the death event occurred earlier then 28 days. The following conditional survival curves for five patients showing how survival probability varied with the marker. The solid survival curves represent the median estimator and the corresponding longitudinal trajectories are depicted in the dotted boxes at the bottom left with four measurements because these patients discharged or died before the 28-day since admission to intensive care wards

Of 280 patients in this cohort, 112 (40%) patients died at 28-day since admission. One hundred thirteen patients received respiratory support during the disease course and among which 107 (94.7%) died at 28-day. Basically, the dynamic profile of SpO_2_/FiO_2_ measurement (square root) was more stable and presented with a rising trend in survivors as compared to non-survivors, and we also observed a sharply decrease of SpO_2_/FiO_2_ over the first few days for non-survivors (Fig. 1). From the developed joint model (Table 1), in addition to the fact that older age, lower lymphocytes count, and higher content of D-Dimer at baseline could pose an unfavorable effect to prognosis of intensive care patients with COVID-19, we also observed a strong and significant association between the square root SpO_2_/FiO_2_ value and the risk for death, with a unit decrease in the marker corresponding to 1.82-fold increase in the mortality risk (95% CI: 1.56–2.13). We then took five patients as examples and focused on the conditional survival probabilities at day 28 (Fig. 1).

- Patient A showed a slightly decreased SpO_2_/FiO_2_ with a descending conditional survival, but the condition was improved along with a dramatic elevation of the marker at the forth measurement. This patient did not receive any respiratory support and discharged at day 27 after admission.
- Patient B who showed a stable SpO_2_/FiO_2_ had a higher survival chance of not experiencing death; the patient did not receive any respiratory support and discharged at day 16 after admission.
- Patient C showed a deteriorated respiratory condition regarding the decreasing trend of SpO_2_/FiO_2_, and died at day 17 after admission. The corresponding conditional survival was declined over time but showed a slightly condition improvement afterwards, which was also reflected as the value of SpO_2_/FiO_2_ increased from the third to the forth measurement. This patient received invasive mechanical ventilation for respiratory support.
- Patient D showed a fluctuant level of SpO_2_/FiO_2_ and died at day 14. Consistent with the unstable respiratory condition, the conditional survival changed over time and led to an unfavorable prognosis. This patient received non-invasive mechanical ventilation for respiratory support.
- Patient E showed an increasing value of SpO_2_/FiO_2_ marker from the first to the forth measurement, indicating an improvement of condition. No respiratory support was given and the patient discharged at day 16 after admission.

**Table 1:** Summarization of the joint longitudinal-survival model

	Joint Model	
	Coefficient (95% CI)	***P***
***Longitudinal process (Linear Mixed-effects model)***		
Day	0.017 (0.0054, 0.0286)	0.0040
Respiratory support	−1.138 (− 1.2697, − 1.0063)	< 0.0001
Day: Respiratory support	−0.0767 (− 0.0981, − 0.0553)	< 0.0001
***Event Process (Weibull relative risk model)***		
Age	0.047 (0.028, 0.066)	< 0.0001
Lymphocytes	−1.1542 (− 1.7099, − 0.5985)	< 0.0001
D-Dimer	0.0332 (0.0179, 0.0485)	< 0.0001
Association^a^	−0.6012 (− 0.7547, − 0.4477)	< 0.0001

^a^Association between true measurements of SpO_2_/FiO_2_ marker and mortality risk

## Discussion

Previous studies manifested the applicable value of SpO_2_/FiO_2_ in acute respiratory distress syndrome (ARDS) and acute hypoxemic respiratory failure [5, 6], but evidence is limited for COVID-19. Continuous pulse oximetry has been incorporated into standard monitoring in the intensive care unit for decades. Use of the pulse oximetry to monitor the SpO_2_/FiO_2_ for intensive care patients has many advantages. First, the noninvasive nature of pulse oximetry avoids excessive arterial blood draws which are painful. Second, compared to intermittent sampling of arterial blood gas, pulse oximetry allows continuous monitoring of the oxygen saturation, which may increase the likelihood of early detection of ARDS.

Utilizing a more informative joint model, we demonstrated the prognostic value of SpO_2_/FiO_2_ for intensive care patients with COVID-19 where its decreasing trajectory is tightly associated with an increasing risk of mortality. Clinically, many factors that affect the progression of the disease (i.e., pulmonary or non-pulmonary infections, potential lung injury, surgery) may cause changes in SpO_2_/FiO_2_ objectively. To be specific, pulmonary infection may affect oxygenation state, resulting in a decrease in peripheral SpO_2_. At this time, SpO_2_/FiO_2_ will continue to decline if no sufficient oxygen concentration was supplied by respiratory support [7]. Additionally, human factors such as unstandardized time frequency of sampling and measurement may also change the value of SpO_2_/FiO_2_ subjectively.

We acknowledge limitations of this study. First, few patients undergone arterial blood gas sampling in our cohort which means hardly can we compare the predictive performance of SpO_2_/FiO_2_ to PaO_2_/FiO_2_. Second, the duration and mode of respiratory support and the positive end-expiratory pressure which are known to be particularly relevant to the ratio of SpO_2_/FiO_2_, were not recorded and may cause bias when profiling the longitudinal outcome. Third, potential confounders such as the time of hospital staying, speed in recovery and intensive care upgrade which might be probably informative were not considered due to a substantial missing data.

In summary, since pulse oximetry is continuously available, the abovementioned advantages coupled with data from the current study suggested that SpO_2_/FiO_2_ could serve as a non-invasive prognostic marker in intensive care patients with COVID-19 to facilitate early adjustment for treatment, thus improving overall survival.

## Data Availability

Dr. J. Wang had full access to all of the data in the study. After publication, the data will be made available to others on reasonable requests after approval from the corresponding author (J.W, dr_wangjun@suda.edu.cn) and Wuhan Tongji Hospital.

## Abbreviations

COVID-19: Coronavirus disease 2019
SpO_2_/FiO_2_: The oxygen saturation to fraction of inspired oxygen ratio
PaO_2_/FiO_2_: The arterial oxygen partial pressure to fractional inspired oxygen ratio
